# A union of deep learning and swarm-based optimization for 3D human action recognition

**DOI:** 10.1038/s41598-022-09293-8

**Published:** 2022-03-31

**Authors:** Hritam Basak, Rohit Kundu, Pawan Kumar Singh, Muhammad Fazal Ijaz, Marcin Woźniak, Ram Sarkar

**Affiliations:** 1 Department of Electrical Engineering, Jadavpur University, 188, Raja S.C. Mallick Road, Kolkata, West Bengal 700032 India; 2 Department of Information Technology, Jadavpur University, Jadavpur University Second Campus, Plot No. 8, Salt Lake Bypass, LB Block, Sector III, Salt Lake City, Kolkata, West Bengal 700106 India; 3 Department of Intelligent Mechatronics Engineering, Sejong University, Seoul, 05006 Korea; 4 Faculty of Applied Mathematics, Silesian University of Technology, 44-100 Gliwice, Poland; 5 Department of Computer Science & Engineering, Jadavpur University, 188, Raja S.C. Mallick Road, Kolkata, West Bengal 700032 India

**Keywords:** Computational science, Computer science, Information technology, Software

## Abstract

Human Action Recognition (HAR) is a popular area of research in computer vision due to its wide range of applications such as surveillance, health care, and gaming, etc. Action recognition based on 3D skeleton data allows simplistic, cost-efficient models to be formed making it a widely used method. In this work, we propose *DSwarm-Net*, a framework that employs deep learning and swarm intelligence-based metaheuristic for HAR that uses 3D skeleton data for action classification. We extract four different types of features from the skeletal data namely: Distance, Distance Velocity, Angle, and Angle Velocity, which capture complementary information from the skeleton joints for encoding them into images. Encoding the skeleton data features into images is an alternative to the traditional video-processing approach and it helps in making the classification task less complex. The Distance and Distance Velocity encoded images have been stacked depth-wise and fed into a Convolutional Neural Network model which is a modified version of Inception-ResNet. Similarly, the Angle and Angle Velocity encoded images have been stacked depth-wise and fed into the same network. After training these models, deep features have been extracted from the pre-final layer of the networks, and the obtained feature representation is optimized by a nature-inspired metaheuristic, called Ant Lion Optimizer, to eliminate the non-informative or misleading features and to reduce the dimensionality of the feature set. DSwarm-Net has been evaluated on three publicly available HAR datasets, namely UTD-MHAD, HDM05, and NTU RGB+D 60 achieving competitive results, thus confirming the superiority of the proposed model compared to state-of-the-art models.

## Introduction

Human Action Recognition (HAR)^1^ is a highly dynamic and peremptory research area in the domain of image and video processing. It refers to the automated identification of the actions of one or many subjects through a sequence of observations. The automatic interpretation of human-environment interaction has been a vital domain for research due to its promise in both online and offline applications viz., visual surveillance, gaming, automatic video annotation, assisted living, automation-based driving, and health monitoring^2^.

Deep learning is very effective for classification problems, where related tasks benefit from each other (transfer learning) since it performs end-to-end optimization. Several recent action recognition methods^3–5^ employ deep Convolutional Neural Networks (CNNs), Recurrent Neural Networks (RNNs) and Graph Convolutional Networks (GCNs)^6,7^ for achieving commendable results in both 2D and 3D HAR problems. The drawback of using RNN is that all spatial dependencies are not captured by RNNs, since information about the spatial structure of the skeleton in 3D HAR requires the knowledge of a specific traversal of the joints. GCNs have scalability issues concerning their number of nodes, and hence may be proven insufficient for complex 3D HAR tasks.

To convert the HAR task into an image classification problem, image encoding of features is a popular methodology^8^ that converts the 3D skeletal data into images. Image Encoding techniques extract geometrical features from the key joints of the skeletons. The drawbacks of the existing image encoding practices include the insensitivity of features from highly localized movements, features’ fusion from the same orthogonal planes, the lack of motion information, and the high number of channels in the encoded image thus increasing the computational cost.

Several methods exist for HAR from RGB videos (2D action recognition) like^9,10^, but the drawback of harnessing only the RGB modality is the high level of abstraction and the difficulty in handling the temporal dimension. Besides, the classification task from RGB video is sensitive to multiple factors like viewpoints, the background, and illumination conditions. The 3D convolution frameworks have also been proposed in recent studies like Cao et al.^11^, Papadopoulos et al.^12^ some of which show promising results.

With developments in multimedia computing like the introduction of economical Kinect depth sensors (Microsoft Kinect^13^, for example), extracting skeletal data has been made easier. In HAR, skeletal representation refers to the set of points in 3D space, each of which indicates the physical position of a specific joint in the subject’s body. In comparison to 2D HAR tasks, 3D HAR uses skeletal details as the primary source of input information^14^, since such representations are more resilient to challenges such as dynamic video conditions and body parts’ occlusion that were prevalent in the 2D HAR tasks.

**Figure 1:**
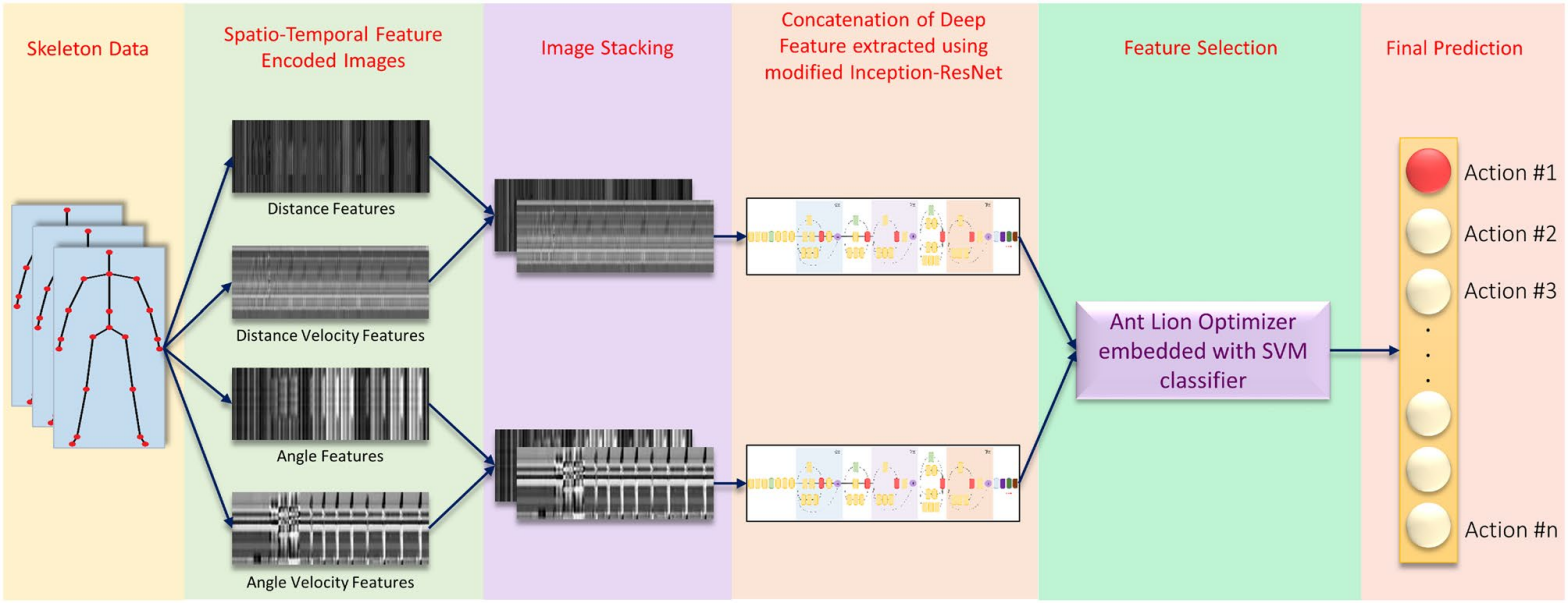
Overall workflow of the proposed DSwarm-Net model for solving 3D HAR problem.

CNN is a widely explored and one of the most popular deep learning-based tools for HAR task due to its better representational abilities than the RNNs. However, processing the RGB or depth modality of the action data through CNN requires huge computational cost and memory requirements which might not be scalable in the real world. To address this challenge, in the present work, we propose a novel method, called DSwarm-Net, involving a swarm intelligence-based optimization algorithm along with the CNN framework for the HAR task. First, we extract four distinctive spatio-temporal feature vectors from the relative movements of the skeletal joints. These features are thereafter encoded into images, which are fed to the CNNs for deep feature extraction. We also employ Ant-Lion Optimization (ALO) to remove redundant and misleading information from the feature space. Finally, we use a classification head to have the final prediction of actions. The overall workflow of our proposed HAR framework is shown in Fig. 1.

## Literature survey

Multi-modal approaches for HAR have been used in literature^15–17^, but 3D HAR is generally based on skeletal data^14^ unlike video-based HAR. Spatio-Temporal LSTM networks are widely applied in action recognition tasks, that use a gating mechanism^18^, or attention mechanism^19^.

Devanne et al.^20^ used a depth sensor for extracting a compact representation of an action class and developed a fitting algorithm to use the 3D coordinates of the joints in the consecutive frames as a trajectory. Then they used a Riemann manifold to compute the similarity of trajectory and a KNN classifier for final classification. Liu et al.^21^ proposed a unified framework for both single and multiple-view action recognition. They use a hierarchical partwise bag-of-words representation to encode local and global features and then formulate a part-regularized multitask structural learning framework for action classification. Hou et al.^22^ used skeleton data for encoding the spatial-temporal information into colour texture images and fine-tuned a pre-trained CNN model in three different ways: using front, top and side views. The decision scores of the three models were fused using the late score fusion technique for the final classification. On the UTD MHAD dataset, their proposed method got an accuracy of 86.97%.

Yang et al.^23^ proposed a two-branch attention model that attends to the key stages of Spatio-temporal features, filtering out the misguiding joint predictions. They combined their two-branch attention network with a “Sub-Sequence Attention Network” for improving the performance. They achieved 82.4% accuracy on the NTU RGB+D 60 dataset on cross-subject classification. Mansur et al.^24^ used dynamic features extracted by applying inverse dynamics to a physics-based representation of the human skeleton. They used a low-dimensional feature representation with a hidden Markov classification framework. Yang et al.^25^ proposed a latent max-margin multi-task learning model that uses 3D skeleton data for action classification. They take into consideration the intrinsic inter-dependencies between the joints in the human skeleton and the action classes leading to improved performance.

Recently GCNs have been implemented in the task of HAR as shown by^26,27^ where the skeleton sequences are considered as graphs and the Spatio-temporal features are captured by the GCNs whereas Song et al.^28^ showed that fusion of multiple GCNs can even improve the classification performance. Shi et al.^29^ used two-stream adaptive GCNs where the topology of the graphs can be either learned by the end-to-end Backpropagation algorithm or by uniform learning with an additional benefit of flexibility for graph construction. Yang et al.^30^ introduced pseudo-GCN, where a learnable matrix was introduced instead of a fixed adjacency matrix. Thus the network learns dependencies among joints well as well as captures deep multi-level features followed by a hybrid Spatio-temporal multi-level attention module to produce comparable results with the state-of-the-art results on HDM05 and NTU RGB+D 60 datasets. Recently, Liu et al.^31^ proposed a unified spatio-temporal graph convolutional operator to disentangle multi-scale graph convolutions to address the research gap of unobstructed cross-spacetime information flow and unbiased long-range joint relationship modeling under multi-scale operators. However, all these approaches suffer from the shortcomings of exceeding computational complexity and inflexible receptive fields in the spatial and temporal graphs. To alleviate the problem, Cheng et al.^32^ proposed Shift-GCN, consisting of shift graph operations and lightweight point-wise convolutions. The performance of Shift-GCN notably exceeds the state-of-the-art methods with 10X fewer parameters. LSTM based networks such as^33^ have also been implemented recently to remove the intra-class diversity by adapting a Spatio-temporal auto-encoder, although they have failed to accomplish comparable results with the GCN based approaches, thereby proving the superiority of GCN over LSTM networks.

ALO^34^ is a widely used optimization algorithm, based on the behaviour of the ants and the antlions in nature. Recently, it has been applied to several domains including engineering applications, power systems, economic load dispatch, PID controller, and many more. For example, Heidari et al.^35^ proposed an efficient training algorithm for solving the optimal multi-layer neural network problem. Here ALO, as compared to other population-based and heuristic optimization algorithms, performed superior in terms of convergence ability. Ali et al.^36^ used the ALO for finding a solution to minimize the whole running time of the Directional Over Current (DOC) relays. The authors used ALO particularly for determining the optimal location of the DG unit in the Radial Distribution Network (RDN). Optimal Reactive Power Dispatch (ORPD) is an important economic load dispatch problem and can be achieved through the determination of an optimal set of reactive compensation devices, transformer turns ratio, etc. Mouassa et al.^37^ successfully used ALO in this particular task, achieving the minimum transmission loss and optimal set of ORPD parameters. Besides, ALO has been used in several other applications like optimal design problem^38^, smart grid design^39^, image processing^40^, networking applications^41^, etc. These applications establish the versatility and usefulness of the ALO in solving different complex optimization problems.

## Methods

In this paper, we propose a deep learning-based method that uses skeleton data for the 3D HAR task. More specifically, the proposed approach extracts four types of informative features (Distance, Distance Velocity, Angle, and Angle Velocity features) from the 3D skeleton data, and encodes them into images using a suitable encoding scheme. The Distance and Distance Velocity features based images have been stacked (depth concatenation) to form “compact distance inputs” and Angle and Angle Velocity feature-based images have been stacked to form “compact angle inputs”. These compact inputs have been fed to a CNN architecture customized by us inspired by the Inception-ResNet^42^ architecture. The CNN model is trained twice separately (from scratch)^43^ for both types of compact inputs and 2048 deep features are extracted from the pre-final layer of each CNN. These deep features have been concatenated to form a 4096 sized feature space, which has been fed to the ALO for feature selection, embedded with an SVM classifier for fitness evaluation in the ALO and final predictions.

### Spatio–temporal feature extraction

**Figure 2:**
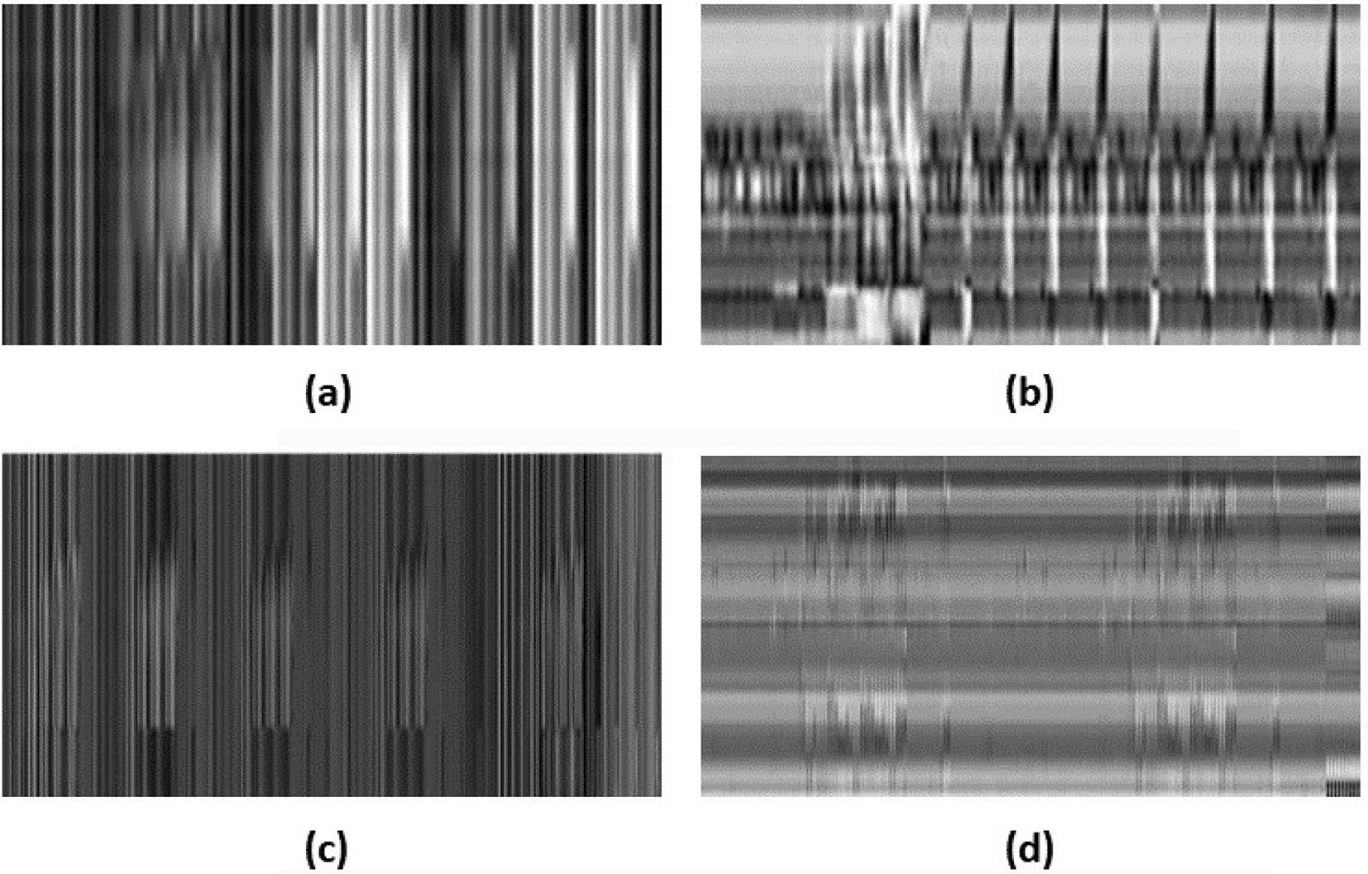
Encoded gray-scale images generated from four different features: (**a**) distance (**b**) distance velocity (**c**) angle, and (**d**) angle velocity features obtained from the UTD-MHAD dataset. Angle features contain more expressive information as compared to distance features.

Instead of the previous methods that used the 3D skeleton information to project on three orthogonal planes, we have extracted four different types of features and mapped them in the form of two-dimensional encoded images. The feature extraction methods and their pre-processing are described in the following sections. Figure 2 contains the representative encoded grayscale images for four different feature vectors.

#### Distance features

Following the work of^44^ the distance features, which contains important relative spatial information about the joints, are computed by measuring the separation between any two fixed joints. Consider a skeleton-based representation has N joints and M frames. For $i^{th}$ joint (where $i\in N$) in each frame, let the position vector be $p_{i}=\{p_{i}^{x},p_{i}^{y},p_{i}^{z}\}$. So, for $m^{th}$ frame (where $m\in M$), we have *N* such position vectors $P_{m}=\{p_{1}, p_{2}, p_{3}, \ldots , p_{N}\}$ and for the entire M video frames, we have $P=\{P_{1}, P_{2}, P_{3}, \ldots , P_{M}\}$, consisting of spatial location of human-body joints having the dimension of $M \times N \times 3$.

If we consider the $j^{th}$ frame, the Euclidean distance between the joints k and l (where $k\ne l$) is defined as:

1 $$\begin{aligned} d_{kl}^i=||p_k^j-p_l^j||_2 \end{aligned}$$

Therefore the joint distance features, arranged in increasing temporal order, from a skeleton sequence, is defined as:

2 $$\begin{aligned} DF_m=\{ d_{kl}^1, d_{kl}^2, \ldots , d_{kl}^m\} \end{aligned}$$

Thus we represent the temporal features explicitly whereas the spatial configuration of joints can be expressed implicitly through the pair-wise distances. Then we scale the number of columns of *DF* from *m* to $m'$ using bilinear interpolation.

3 $$\begin{aligned} DF=I(DF_m)=\{d_{kl}'^1, d_{kl}'^2, \ldots , d_{kl}'^{m'}\} \end{aligned}$$

where, $I(DF_{m})$ is the interpolation function.

#### Distance velocity features

The temporal difference between the distance features as described above is captured by the Distance Velocity Features (DVF) vector. Du et al.^45^ proposed a similar approach wherein they used the marker coordinates of the raw skeleton. However, the distance velocity features extract information about the kinematic pattern of the skeleton sequences. For capturing the spatial and temporal information, the temporal difference over the distance feature vectors has been used. This method is especially useful when extracting features from classes that have a rapid spatial movement, like *Jog* or *Run*. The temporal motion of the classes and their spatial characteristics are equally important, the span and direction of which are used as information sources.

The DVF for an interval $\omega$, can be expressed as Equation 4, for $t \in \tau$ and $t< \tau -\omega$, $\tau$ being the total time of the video sequence.

4 $$\begin{aligned} DVF_{t}=DF_{t}-DF_{t+\omega } \end{aligned}$$

And across the whole sequence of the video, we have the distance velocity features as in Equation 5.

5 $$\begin{aligned} DVF=\{DVF_0, DVF_1, DVF_2, \hdots , DVF_{\tau -\omega }\} \end{aligned}$$

#### Angle features

Angle features are defined as the angle between any three skeleton joints from the sequence of the skeleton and contain more discriminating information for some of the special tasks like running, throwing, waving, etc. Unlike the distance features, the angle features change more drastically.

For every frame, if we have joints *a*, *b*, *c* where $\{a,\,b,\,c\}\in M$, *M* being the total number of joints and $a\ne b\ne c$ having angular matrix can be obtained from $p_a,\,p_b\text { and} \ p_c$ which is given by the Equation 6.

6 $$\begin{aligned} \theta _{abc}=cos^{-1}\left( \frac{p_{ab}\cdot p_{bc}}{||p_{ab}||\,||p_{bc}||}\right) \end{aligned}$$

Thus, for $n^{th}$ frame and for *M* number of joints, we can extract $\frac{^M C _3}{2}$ features in the form

7 $$\begin{aligned} AF_n=\{\theta _{a,b,c}\}\,\text { where }\{a,b,c\}\in [0,N] \end{aligned}$$

So, for the entire sequence, we have the angle features as follows:

8 $$\begin{aligned} AF=\{AF_n\}\text { where }n=[0,M] \end{aligned}$$

#### Angle velocity features

The angle velocity features (AVF) vector representation is important for classes that have a significant angular movement like *Arm Curl* or *Draw Circle*. It can discriminate between these specialized movements with ease. The AVF can capture the temporal difference in an inter-frame scenario, which is similar to the concept of Optical Flow in 2D HAR tasks^46^.

The AVF for an interval $\omega$, can be expressed as Equation 9, for $t \in \tau$ and $t< \tau -\omega$, $\tau$ being the total time of the video sequence, and *AF* being the Angle Features, as described in the previous subsection.

9 $$\begin{aligned} AVF_{t}=AF_{t}-AF_{t+\omega } \end{aligned}$$

Across the whole sequence of the video, we have the angle velocity features as given in Equation 10.

10 $$\begin{aligned} AVF=\{AVF_0, AVF_1, AVF_2, \hdots , AVF_{\tau -\omega }\} \end{aligned}$$

### Image encoding of extracted features

Skeleton data are considered as five-dimensional (5D) data points that include three coordinate dimensions, a time label and a joint label. Hence, we have transformed the sequence to visualize them as a series of RGB images that encodes Spatio-temporal information of skeleton data descriptively. This image encoding scheme makes the data more compatible and enables the CNN to learn discriminative features from the skeleton data. Following the original work of^45^, we can convert the sequence of features

11 $$\begin{aligned} F=\{F_n\}\,\text { where }n\in [0, \tau ] \end{aligned}$$

to the intensity mapping of the encoded image by using the following transformation:

12 $$\begin{aligned} I_{i,j}=\lfloor (255-0)\times \frac{(F_{i,j}-\min (F_i))}{\max (F_i)-\min (F_i)}\rfloor \end{aligned}$$

where, $I_{i,j}$ is the pixel intensity of the index *(i,j)* in the encoded image where, *i* is the number of frame of the video sequence and *j* is the number of feature, $F_{i,j}$ is the $j^{th}$ feature in the $i^{th}$ frame and $F_i$ is the $i^{th}$ frame in the feature sequence *F*.

Unlike the original work of Du et al.^45^, who have used the image encoding to map those features in three orthogonal planes of RGB image, we have used this scheme to frame the complementary features in single-channel grayscale images similar to Fig. 2. Besides, we have mapped the information of a frame from the sequence into a single row of the encoded image unlike^45^ to ensure that the extrema used in the equation above are not affected by the presence of global outliers across the frame. The reason behind the channel modification is that classification of a single channel grayscale image calls for lesser memory allocations and fewer parameters with similar performance as compared to three-channel RGB images. Thus, by preserving frame-wise locality, we encode the features into images of dimension $m\times n$ where *m* is the number of frames in a single video sequence and *n* is the number of features extracted from that frame. In the case of videos having different frame lengths, i.e. different *m* values, the encoded images from these video sequences having dimension $m\times n$ are further resized to a fixed dimension of $m_0\times n$ using bicubic interpolation, where, $m_0$ is the fixed value of frame length in a video sequence.

### Compact distance and angle encoding

The feature encoded images extracted from the skeleton of the datasets have dimensions as follows:

$$\begin{aligned}&Distance: 65\times 190\times 1\\&Distance\; Velocity: 65\times 190\times 1\\&Angle: 70\times 3420\times 1\\&Angle\; Velocity: 70\times 3420\times 1 \end{aligned}$$

In the present work, image stacking has been performed by concatenating the images depth-wise, leading to separate images which are of size $l\times b\times h_1$ and $l\times b\times h_2$, to now be a single image of size $l\times b\times (h_1+h_2)$. On the Distance and Distance Velocity feature-encoded images, this method leads to “compact distance inputs” of size $65\times 190\times 2$. Similarly, applying depth concatenation on the Angle and Angle Velocity encoded images results in a “compact angle inputs” of dimension $70\times 3420\times 2$.

Such compact encoding results in only two types of inputs and thus two CNN architectures are used rather than using four CNN classifiers for the four types of feature-encoded images, thereby decreasing the computation cost. Channel-wise aggregation tends to lose information about the input and thus image stacking is preferred here since the important features will be learned by the deep learning model while rejecting the redundant information. Attempting to decrease the computation cost by aggregating the feature-encoded images leads to higher rates of misclassification for a small decrease in the computation complexity, which is an undesired trade-off in this scenario. So we chose to perform depth concatenation-based image staking thus preserving the feature information rather than saving the computation cost, to ultimately obtain superior performance. Additionally, the angle and angle velocity features contain more refined and edifying information of the relative movement of the body joints. Encoding those features into images results in a high-dimensional image as compared to the distance features, and therefore, we have only merged the encoded images with the same dimensions, alleviating the requirement of reshaping, thereby limiting the loss of potentially important information.

**Figure 3:**
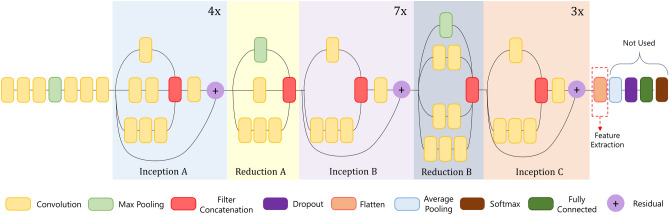
Architecture of the proposed CNN used in this study.

### CNN for deep features extraction

Traditional machine learning methods extract hand-crafted features which need to be selected manually, and thus non-informative features may get selected making the classification erroneous. Deep Learning on the other hand learns the essential feature set on its own using backpropagation and thus is more feasible. So, in the development of this framework, we use extract deep features instead of machine learning features.

The proposed method for mapping skeletal data to images like tensors makes it flexible to experiment with a wide variety of previously deployed and potential future CNN architectures. As the processing of images is quite easier as compared to skeleton sequence, temporal image encoding reduces the work from skeletal data processing to simple image classification tasks, with all the skeleton information embedded in the encoded images. In the proposed DSwarm-Net model, we use a modified Inception-ResNet^42^ architecture for the image classification task with remarkable efficiency. The Inception-ResNet network has been previously successfully applied on tasks like face recognition^47^. Besides, the Inception-ResNet architecture allows us to experiment with different input image specifications, both in terms of the number of channels and spatial image dimension. Therefore, we have used it as the test-bed for our experiments of extracting features from encoded skeleton images.

Being inspired by the original network architecture, we have made substantial changes in the layers of the network to serve our purpose. Figure 3 shows the architecture of the CNN used for feature extraction. Categorical cross-entropy loss is used for this specific purpose, and model accuracy and losses are monitored over the iterations, and the weights for the best accuracy model are saved for further use. Finally, 2-channel encoded distance and angle encoded tensors are feed-forwarded through the CNN and the outputs from the penultimate dropout layer are extracted. In Fig. 3, the fully connected layers have been shown for the CNN model. These fully connected layers have been used during the training of the CNN model using the encoded images and the corresponding class labels. After the feature learning process, the fully connected layers have been removed to extract the final feature set from the penultimate layer of the model to be optimized using ALO in the next step of the framework.

Following the original work^42^ for the training of the CNN, which demonstrates the compatibility of the CNN used with DSwarm-Net, we started the training from scratch. We have conjectured that training the network from scratch, rather than fine-tuning, resulted in a more superior manner, probably due to the difference in visual patterns, as compared to natural scene images. We have stacked angle, angle-velocity features, and distance, distance-velocity features separately and created two parallel CNN for classification of these 2-channel tensors, and thus by depth-concatenation, the number of parallel CNN branches have been reduced from four to two, bringing down the memory requirements.

To demonstrate the additional benefits of data augmentation with the distance and velocity encoded images, we have also trained the proposed CNN with the augmented dataset, which effectively reduces the possibility of model over-fitting. Some of the popular data augmentation methods like vertical or horizontal flipping fail in this task because the patterns present in the encoded images are distorted by these augmentation strategies, thereby changing the labels of encoded images. So, as a remedy, we have used the additive Gaussian noise for the data augmentation strategy, which effectively doubles up the training data volume. Samples were drawn from zero to mean Gaussian distribution with the standard deviation value set as 0.02. Finally, it was added to the skeleton frame sequence, with the observation that minor variations of the skeleton position or velocity information do not effectively affect the skeleton information much to change the label of the specific action task. This specific data augmentation strategy already gained significant superiority in performance over the existing methods, removing the necessity of further data augmentation strategy.

After processing the skeletal sequence with the CNN, it becomes more practicable to exploit the extracted features from the pre-final layer of the CNN to further process them. As discussed before, a single encoded image contains spatial information for a single skeletal sequence only, therefore to increase the robustness of the model, the features extracted from the parallel CNNs are further processed through linear concatenation before feeding it to the ALO for feature selection and final classification.

### Feature selection using ant lion optimizer

End-to-end classification using deep learning frameworks requires a large amount of data, which is often unavailable in HAR tasks, where there is a large number of classes in the dataset with very few samples belonging to each class. This hinders the optimal performance of CNN models. Also, directly processing skeleton data demands a large number of computational resources since a large amount of information, all of which might not be discriminative is present. When the number of features in a classification model becomes very large, it is preferable to reduce the dimension of the feature set before the final classification. This is because, in a large feature space, redundant as well as deceptive features might be present that may lead to degraded classification performance. Feature selection procedures^48–50^ help eliminate these non-informative and misleading features and further lowers the storage requirement of the machine and leads to faster and better performance of the classification algorithm. Hence for this purpose, we use an evolutionary meta-heuristic, called ALO, which was proposed by Mirjalili et al.^34^.

**Figure 4:**
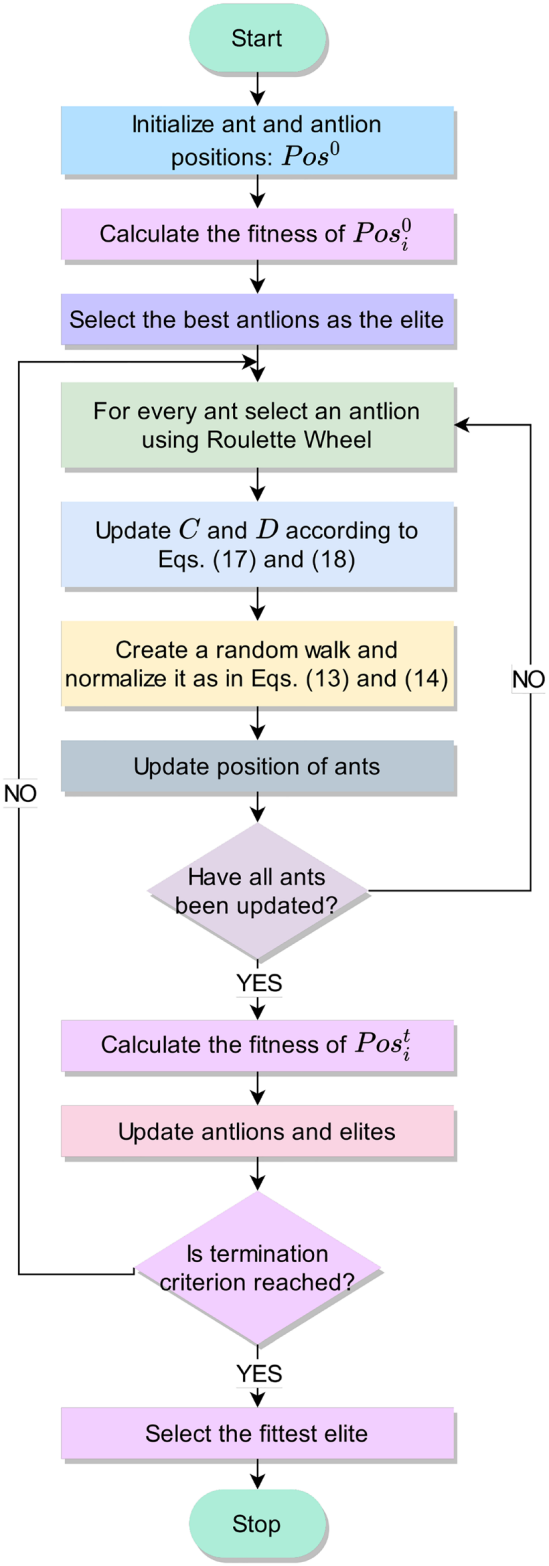
Flowchart of the ALO algorithm used in our proposed DSwarm-Net model.

The ALO algorithm is based on the interaction between the antlions and the ants in a trap. The ants move stochastically in nature for food sources, whereas the antlions position themselves to trap and consume the ants. This movement is affected by the positions of the antlions, who form traps randomly. The randomized movements of the ants can be represented by Eq. (13).

13 $$\begin{aligned} \begin{aligned} Pos_{ant}(t)=[0,cs(2R(i_1)-1),cs(2R(i_2)-1), \ldots , cs(2R(i_T)-1)], \end{aligned} \end{aligned}$$

where, ‘*cs*’ represents the cumulative sum operation, $i_t \mid \, t\in \{1,2, \ldots , T\}$ represents the number of current steps/iterations, where *T* is the maximum number of iterations, *R*(*i*) is a random binary generator that generates 0 or 1 in the $i^{th}$ step. This equation is normalized using min-max normalization and used for updating the positions of ants in the search space, as shown in Eq. (14).

14 $$\begin{aligned} Pos_j^i=\frac{(Pos_j^i-A_j)\times (B_j-C_j^i)}{(D_j^i-A_j)}+C_j, \end{aligned}$$

where, $A_j$ and $B_j$ are the minima and the maximum of the random walks in $j^{th}$ variable respectively and, $C_j^i$ and $D_j^i$ represent the minimum and the maximum of the $j^{th}$ variable respectively, in the $i^{th}$ iteration.

The movements of the ants in the hyperplane are affected by the traps of the antlions. This can be mathematically expressed by Eqs. (15) and (16).

15 $$\begin{aligned} C_k^i= & {} Antlion_k^i+C^i \end{aligned}$$

16 $$\begin{aligned} D_k^i= & {} Antlion_k^i+D^i \end{aligned}$$

Here, $Antlion_j^i$ is the position of the $k^{th}$ antlion in the $i^{th}$ iteration, $C^i$ indicates the minimum of all variables in the $i^{th}$ iteration, $D^i$ represents the vector including the maximum in the $i^{th}$ iteration, $C_k^i$ and $D_k^i$ indicate the minimum and the maximum of all variables for the $k^{th}$ ant in the $i^{th}$ iteration, respectively.

With every iteration, we assign fitness and update the positions in the position matrix for every ant and antlion. With the overall mechanism explained so far, antlions can build their trap based on their fitness, i.e., an antlion with more fitness has a higher probability to build a bigger trap and catch ants with more ease. The ALO algorithm utilizes a Roulette Wheel mechanism for selecting antlions based on their fitness. To capture the ants inside the trap, the antlions throw sand inside the pit. This can be explained mathematically by Equation (17) and Equation (18).

17 $$\begin{aligned} C^i= & {} \frac{C^i}{I} \end{aligned}$$

18 $$\begin{aligned} D^i= & {} \frac{D^i}{I} \end{aligned}$$

where, $I= 10^{\alpha }\times \frac{i}{T}$, where, *i* is the current iteration, *T* is the maximum number of iterations, and $\alpha$ is a constant such that:

19 $$\begin{aligned} \alpha ={\left\{ \begin{array}{ll} 2 &{} \text {when }t>0.1T\\ 3 &{} \text {when }t>0.5T\\ 4 &{} \text {when }t>0.75T\\ 5 &{} \text {when }t>0.9T\\ 6 &{} \text {when }t>0.95T \end{array}\right. } \end{aligned}$$

where, *t* is the current iteration and *T* is the total number of iterations. So, based on Equation 17 and Equation 18, the radius of updating ant’s position is decreased at every iteration and it mimics the sliding of the ants inside the trap. Finally, when the fitness of an ant is greater than or equal to the fitness of the antlion, it is assumed that the antlions have consumed the ant, and the antlion moves to the position of the fitter ant. This can be explained mathematically by Equation 20 where, $Antlion_k^i$ and $Ant_k^i$ represent the positions of $k^{th}$ antlion and ant respectively in the $i^{th}$ iteration.

20 $$\begin{aligned} \begin{aligned} Antlion_k^i=Ant_k^i, \, \text { if }fitness(Ant_k^i)\ge fitness(Antlion_k^i) \end{aligned} \end{aligned}$$

The ALO algorithm was traditionally proposed to solve continuous optimization problems. Thus, to make the algorithm compatible with feature selection tasks, we map the continuous search of ALO to binary search. A Sigmoid-shaped function is used as a transfer function to convert the continuous values to binary encoded candidate solutions, as shown by Equation 21.

21 $$\begin{aligned} F(m) = \frac{1}{1+e^{-m}} \end{aligned}$$

The fitness of the ant and antlion population (denoted by *A*) are calculated using Equation 22, where, ‘*acc*(*A*)’ represents the classification accuracy obtained by the candidate solution ‘*A*’; $\alpha$ represents a weighting factor, and ‘*FS*(*A*)’ represents the number of features selected out of *D* total features, by the candidate solution *A*. Thus, the fitness function is a weighted sum of the accuracy and the fraction of features *not* selected by the candidate solution.

22 $$\begin{aligned} fitness(A) = \alpha \times acc(A) + (1-\alpha )\times \frac{D-FS(A)}{D} \end{aligned}$$

The exploration capability of the ALO algorithm due to the randomized antlion selection and the formulation of random walk of the ants around the antlions. The exploitation capability of the ALO is attributed to the shrinking of the boundaries of the antlions’ traps. This makes the ALO algorithm a robust choice for solving the feature selection problem.

The overall workflow of ALO algorithm used for feature selection is described in Fig. 4.

## Results and discussions

In this section, we first describe the datasets used for evaluating the performance of the proposed model and show the results thus obtained. Furthermore, we compare our results with some existing models in literature on the same datasets.

### Description of datasets

We have used the skeleton data only from the datasets for the action recognition task and have discarded other modalities of available data due to reducing extremely high pre-processing and large memory requirements. The specifications of the datasets used are mentioned in Table 1.

**Table 1:** Specifications of the different datasets used in the present work.

Dataset	Number of video sequence	Action classes	Subjects	Keyjoints
UTD-MHAD^51^	861	27	8	20
HDM05^52^	2337	130	5	31
NTU RGB+D 60^53^	56880	60	40	25

#### UTD multimodal human action dataset

The UTD-MHAD dataset^51^ consists of the image sequence of actions performed by 8 different subjects and the data is distributed in 27 different action classes and their corresponding skeleton information. Each of the subjects was recorded four times for each of the 27 actions, resulting in an 861 video sequence in total. For each of the actions, the depth maps, along with RGB, inertial sensors, and skeletal information were recorded, however, for our experiments, we have used the skeleton files only.

Following the work of^54^ we have used the data from odd-numbered subjects (i.e. 1, 3, 5, and 7) for training, and the rest of the data (i.e. subject number 2, 4, 6, and 8) were used for testing purpose, which is known as cross-subject validation protocol.

#### HDM 05

HDM05^52^ is a publicly available motion database that contains more than three hours of systematically recorded and well-documented motion capture data in the C3D as well as in the ASF/AMC data format. Furthermore, HDM05 contains 130 motion classes in 10 to 50 realizations executed by various actors. Classification of action data from this huge dataset is extremely challenging due to the large number of action classes present and their similarity. A different number of repetitions of actions having a high amount of similarity add to the challenge of action recognition from this dataset. To compare our results with the existing methods, we have followed the standard protocol where we have conducted 10 different experiments, each of them sampling the dataset in two equal portions, and utilizing one half for training and the other half for validation and the mean from the experiments was reported.

#### NTU RGB+D 60

The NTU RGB+D 60 dataset^53^ is a large-scale dataset for the HAR task. It contains 56,880 action video sequences, recorded by using 17 different setups, named S001 to S017. The dataset contains videos from 60 different action classes, and these were performed by 40 different human subjects of different age groups, heights, and ethnicity. The 60 action classes are further classified in three V2 cameras concurrently and the dataset contains four different modalities of data for each video sequence: RGB video, depth maps, infrared (IR) videos, and 3D skeleton data, however, in our method, we have used the skeleton data only. Each of the skeleton files contains information about the 25 major body joints of humans whereas the RGB videos have a resolution of 1920$\times$1080.

Following the original evaluation protocol as proposed in^53^, we have performed both the cross-subjects (CS) and cross-view (CV) evaluations, which are done by splitting the dataset into two equal sub-parts containing actions from 20 subjects and by using them for training and testing. One of the major drawbacks of this dataset is that 302 samples had incomplete or missing skeleton data, forcing us to discard those samples for training and testing.

### Implementation

The height configurations $m_0$ of the images are set to 70, 75, and 265, and based on the median length of video frames, the frame interval is set to 5, 5, and 10 for UTD-MHAD, NTU RGB-D 60, and HDM05 datasets respectively. The model is compiled using Adam optimizer with an initial learning rate of 0.001 and categorical cross-entropy loss is implemented for this purpose. $\beta _1$ and $\beta _2$ values are set to 0.9 and 0.999 respectively. $\epsilon$ value is set to 0.01 and the model is fine-tuned by using adaptive learner such that the learning rate is reduced by a factor of 5 upon saturation of accuracy. Batch normalization in the network architecture is found useful for network training and to reduce sensitivity to the initial starting weights. Early stopping is incorporated with a patience value set to 20 epochs to remove the possibility of overfitting. Additive Gaussian noise is found useful for the data augmentation strategy. The model is trained up to 500 epochs with a batch size of 8.

**Table 2:** Classification performance of the proposed DSwarm-Net model on three benchmark HAR datasets. *Acc*: Accuracy, *F*1: F1-Score.

Encoding Used	UTD MHAD		NTU RGB+D 60				HDM05	
			Cross-subject		Cross-view			
	Acc (%)	F1 (%)	Acc (%)	F1 (%)	Acc (%)	F1 (%)	Acc (%)	F1 (%)
Distance encoded	95.62	96	84.49	85	87.24	88	88.45	89
Angle encoded	96.81	97	84.92	85	88.39	89	89.17	89
Distance velocity encoded	90.23	91	83.44	84	87.15	88	88.34	89
Angle velocity encoded	94.55	95	84.63	85	88.15	89	89.09	90
Compact distance encoded	97.56	98	84.81	85	88.92	89	89.46	90
Compact angle encoded	97.97	98	84.97	85	88.66	88	89.88	90
**DSwarm-Net**	**98**.**13**	**98**	**85**.**45**	**86**	**89**.**98**	**90**	**90**.**67**	**92**

Table 2 shows the performance of DSwarm-Net on three standard benchmark datasets. The results justify that depth concatenation of distance and velocity stacked features significantly improves the classification performance, which is also demonstrated by recent works like^55^ in different applications.

### Comparison with other CNN models

As mentioned before, the classification of grayscale encoded images using existing CNN models, pre-trained on large RGB datasets like ImageNet is not practicable due to the difference in patterns in encoded images as compared to natural scene images. Therefore, we have trained the CNN architecture from scratch on the encoded images for the feature extraction task. We have experimented with different CNN backbones for feature extraction, keeping all other experimental modalities the same. Table 3 shows the comparison of classification accuracy using different CNN models as feature extractors with their respective parameter counts. It is observed that our proposed architecture achieves superiority over all other models in terms of classification accuracy with lesser parameters, resulting from a reduction in the number of channels. It is also observed from the table that, with a slightly higher parameter count as compared to the DenseNet201 architecture (18.4M for DenseNet201 vs. 21.8M for our proposed architecture for compact encoded distance features), DSwarm-Net outperforms the former’s result by a significant margin.

**Table 3:** Comparison in terms of the number of parameters and the classification accuracy of the proposed model with other CNN models on UTD-MHAD dataset.

Model	Compact distance encoded features		Compact angle encoded features	
	No. of parameter	Accuracy (%)	No. of parameter	Accuracy (%)
VGG19	57.9 M	83.45	105.7 M	88.45
VGG16	52.6 M	85.78	97.8 M	91.21
ResNet101	42.7 M	88.94	42.7 M	94.32
Inception v3	24.3 M	93.28	24.3 M	95.77
DenseNet201	18.4 M	92.64	18.4 M	95.24
**DSwarm-Net**	**21.8 M**	**97**.**56**	**21.8 M**	**98**.**33**

### Comparison with other optimization algorithms

**Table 4:** Parameter settings for the comparative meta-heuristic optimization algorithms. *OA* denotes Optimization Algorithms.

OA	Parameter(s)	Value(s)
MVO^56^	Minimum wormhole existence probability (${WEP}_{min}$) Maximum wormhole existence probability (${WEP}_{max}$)	${WEP}_{min}=0.2$ ${WEP}_{max}=1$
PSO^57^	Inertia weight (*I*) Acceleration Coefficients ($C_1, C_2$, $C_3$)	*I* lies in [0, 1] $C_1=2, C_2=2, C_3=1$
BAT^58^	Initial loudness (*A*) Pulse rate (*r*) Minimum frequency ($Q_{min}$) Maximum frequency ($Q_{max}$)	$A=0.5$ $r=0.5$ $Q_{min}=0$ $Q_{max}=2$
GWO^59^	Convergence operator (*a*) Exploration Parameter ($r_1, r_2$)	*a* lies in [2 0] $r_1,r_2$ lies in [0, 1]
WOA^60^	Random number (*p*) Spiral Updating Probability (*b*) Random Search Ability (*r*) Random Encircling ability (*e*)	*p* lies in [0, 1] $b=0.5$ $r = 0.1$ *e* lies in [0, 0.5]
FFA^61^	Randomization Parameter, $(\alpha )$ Attractiveness at r=0, $(\beta _0)$ Absorption coefficient, $(\gamma )$	$\alpha$ = 0.5 $\beta _0$ = 0.2 $\gamma$ = 1.0
MFO^62^	Convergence parameter (*a*) Shape of logarithmic spiral (*b*) Closeness Parameter (*t*)	*a* lies in $[-2,-1]$ $b=1$ *t* lies in $[-1,1]$

**Table 5:** Comparison of results (accuracies in %) obtained by the different optimization algorithms (OAs) for feature selection (FS) in our DSwarm-Net model and that obtained without any FS, by end-to-end CNN model.

OAs	UTD-MHAD	NTU RGB+D 60		HDM05
		Cross subject	Cross view	
Without FS	89.34	79.19	80.63	81.53
MVO^56^	96.26	84.67	89.16	90.41
PSO^57^	96.47	83.85	87.58	88.56
BAT^58^	95.74	84.01	88.52	87.42
GWO^59^	95.63	84.43	87.51	88.13
WOA^60^	95.26	84.22	88.13	86.87
FFA^61^	95.84	84.11	87.98	86.55
MFO^62^	95.53	83.89	89.51	89.93
**ALO**	**98**.**13**	**85**.**45**	**89**.**98**	**90**.**67**

For the feature selection step of the DSwarm-Net mode, ALO has been used, and to prove its efficacy over other algorithms, we implemented 7 different optimization algorithms (both classical and popular) for feature selection and classification. The algorithms used for comparison with ALO are:

1. Mean Variance Optimization (MVO)^56^
2. Particle Swarm Optimization (PSO)^57^
3. Bat Optimization Algorithm (BAT)^58^
4. Grey Wolf Optimizer (GWO)^59^
5. Whale Optimization Algorithm (WOA)^60^
6. Firefly Algorithm (FFA)^61^
7. Moth-Flame Optimization (MFO)^62^

Table 4 shows the parameters set for each of the optimization algorithms used for comparison. All of the parameters hold their usual meaning, as referred to in the original papers cited. The same number of population and iterations were used throughout for all the OAs to maintain consistency. Table 5 shows the feature selection performance of ALO as compared to other feature selection algorithms. We can observe from this table that ALO can efficiently reduce the feature dimensionality and boost classification performance in comparison with others. From Table 5 it is observed that without selecting optimal feature set can lead to sub-standard classification performance (as shown in row 1 of Table 5) due to the presence of misleading deep features extracted by the CNN, thereby substantiating the importance of optimal feature selection, leading to comparable results of end-to-end classification performance as shown in Tables 6, 7, and 8 for UTD-MHAD, HDM05 and NTU RGB+D 60 datasets respectively.

### Comparison with existing models

Several methods have been proposed in the recent past to address the HAR problem, and in Tables 6, 7, and 8, we show the performance comparison of our proposed DSwarm-Net model with some of these methods.

**Table 6:** Comparison of our DSwarm-Net model with some recent models on the UTD-MHAD dataset by cross-subject analysis.

Method	Year	Accuracy (%)	Skeleton data	RGB data	Inertial data
Action machine^63^	2018	92.50	Yes	Yes	No
PEM^64^	2018	94.51	Yes	Yes	No
BHDM^65^	2019	92.80	Yes	No	No
Correlation Congruence^66^	2019	94.87	Yes	Yes	Yes
Gimme DSE^67^	2020	93.30	Yes	No	Yes
Fuzzy CNN fusion^68^	2020	97.91	Yes	No	No
SAKDN^69^	2021	98.04	Yes	Yes	Yes
Edge Motion^70^	2021	95.59	Yes	No	No
AMGC^71^	2021	95.11	Yes	No	No
**DSwarm-Net**	**2021**	**98.13**	**Yes**	**No**	**No**

From Table 6, it is clear that the proposed DSwarm-Net performs better than any other existing methods that have been proposed recently. By using skeleton data only, the proposed method outperforms^63,64,67,70,71^ that use skeleton data for action classification. Despite using RGB data, the authors of^63,64,66,69^ rely on temporal dynamics mostly and hence cannot classify between action-classes like *walk* and *jog* with very subtle differences. Our method also outperforms^68^, that uses a similar image encoding algorithm, and recently developed Bayesian hierarchical dynamic model^65^.

**Table 7:** Comparison of our proposed DSwarm-Net model with existing methods on the HDM05 dataset using 10 random split-mean protocol.

Method	Year	Classification accuracy (%)
HCN^72^	2018	86.51
PB-GCN^26^	2018	88.2
Deep STGC^27^	2019	85.29
2S-AGCN^29^	2019	88.5
PGCN-TCA^30^	2020	86.71
SGCN^73^	2021	85.45
Di-StddNet^74^	2021	82.32
**DSwarm-Net**	**2021**	**90.67**

Table 7 shows the comparison of the proposed method with the existing methods on the HDM05 dataset. Our method outperforms both CNN based models^72,74^ and GCN based methods^26,27,73^. Unlike^26^ which uses local body-part information, our method performs better in terms of accuracy by using CNN and optimization algorithm only.

**Table 8:** Comparison of our DSwarm-Net model with some existing methods on the NTU RGB+D 60 dataset.

Method	Year	Cross-subject accuracy (%)	Cross-view accuracy (%)
STVA LSTM^23^	2019	82.40	89.10
Deep STGC^27^	2019	86.45	84.65
PC Net^75^	2019	85.25	91.37
Shift GCN^32^	2020	90.70	96.5
DS LSTM^33^	2020	77.79	87.44
AGC-LSTM^76^	2020	89.20	95.00
PA-ResGCN-B19^77^	2020	90.90	96.00
MV-IGNet^78^	2020	89.2	96.3
VIDA^79^	2020	79.40	84.10
MS-G3D^31^	2020	91.5	96.2
CTR-GCN^80^	2021	92.4	96.8
EfficientGCN-B4^81^	2021	91.7	95.7
ST-TR^82^	2021	89.91	93.1
**DSwarm-Net**	**2021**	**85**.**45**	**89**.**98**

**Table 9:** Results (based on calculated *p*-values) obtained by performing the McNemar’s test between the ALO algorithm used in this paper and the other popular metaheuristics used for comparison.

McNemar’s test	UTD-MHAD	NTU RGB+D 60		HDM05
		Cross subject	Cross view	
ALO vs. MVO	5.79E−03	4.10E−02	3.55E−02	1.96E−04
ALO vs. PSO	3.31E−02	3.43E−02	9.67E−03	4.31E−02
ALO vs. BAT	1.83E−02	3.98E−03	4.80E−02	4.78E−02
ALO vs. GWO	1.01E−02	1.19E−02	3.49E−02	2.45E−02
ALO vs. WOA	2.79E−02	6.31E−03	8.20E−03	1.85E−02
ALO vs. FFA	3.02E−04	3.76E−02	1.75E−02	2.62E−02
ALO vs. MFO	8.48E−03	1.76E−03	7.58E−03	4.83E−02

**Table 10:** Comparison of results obtained by performing stability test on selected features from ALO algorithm along with other popular metaheuristics used for comparison.

OA	UTD-MHAD	NTU RGB+D 60		HDM05
		Cross subject	Cross view	
MVO	0.308	0.310	0.245	0.309
PSO	0.325	0.407	0.376	0.428
BAT	0.290	0.232	0.445	0.324
GWO	0.341	0.444	0.330	0.315
WOA	0.439	0.453	0.409	0.378
FFA	0.391	0.244	0.281	0.236
MFO	0.382	0.259	0.227	0.282
**ALO**	**0**.**458**	**0**.**473**	**0**.**473**	**0**.**472**

Table 8 compares the performance of our method with the existing models on the NTU RGB+D 60 dataset. It is observed from Table 8 that our method significantly outperforms the LSTM based methods like^23,33^ because of incorporating spatial and temporal features for image encoding and classification, which are sensitive to patterns present in the skeleton sequence data. When compared to methods like^27^, they perform similar to our proposed method by utilizing the topological characterization of the action sequence. When compared to CNN-based methods, our model produces comparable results with^75^. Due to the recent success of graph-based methods for the HAR tasks, we have also included some GCN-based approaches like Shift GCN^32^, PA-ResGCN-B19^77^, MV-IGNet^78^, MS-G3D^31^, CTR-GCN^80^, and EfficientGCN-B4^81^ to maintain fair comparison. It can be concluded from the observation that our proposed method outperforms the LSTM-based approaches, but fails to produce comparable results as the graph-based approaches. However, the computational complexity of these graph-based approaches is typically in the range of 15-25 GFLOPS, which often exceeds 100 GFLOPS as well^32^. This results in slower processing and inference speed, which is a major drawback of these methods and often makes them less scalable for real-world applications.

We observe that for some challenging classes of NTU RGB+D 60 dataset (e.g. Shake Head, Writing, Typing on a keyboard, etc.) our model does not perform well because of abundance in similarity of these classes with some other closely-knit classes in terms of spatial and temporal distribution of skeletal data. The class-wise performance indicates that our proposed method fails to distinguish between classes like *Reading* and *Writing*, *Take off a shoe* and *Wear a shoe*, *Typing on a keyboard* and *Writing*, etc. because of the close correlation of those classes in terms of spatial movement of body joints. To mitigate this problem, we plan to utilize 3D flow information apart from the skeletal data to mine more discriminative features in our future work.

### Statistical test

To statistically analyze the significance of our proposed method, McNemer’s test^83^ is performed between the ALO algorithm used in this method with several other metaheuristic optimization algorithms, popularly used for feature selection. McNemer’s test is a non-parametric test, based on the null hypothesis that states two models to be statistically similar^84,85^. If the obtained *p*-value from the test is $<5\%$, then the hypothesis is negated and it is assumed that the two models are statistically dissimilar. Our experimental analysis of the McNemer’s test is demonstrated in Table 9, where for every scenario, the obtained *p*-value is $<0.05$, justifying our proposed method to be statistically dissimilar to them.

### Stability of selected features

Stability testing of a feature selection method has recently become an important metric for measuring the goodness of an optimization algorithm^86^. An algorithm’s stability in feature selection is usually measured by checking the overlap of features selected over multiple independent runs of the metaheuristic. In this work, we assess the feature selection stability using the Jaccard Index between the pairs of best-fit solutions obtained over 25 independent iterations. The pairwise overlap is thereafter computed, i.e. the percentage of instances where one feature is selected in both the solutions. This computation of pairwise Jaccard Index of the best-fit solutions produces a pairwise stability matrix, which is thereafter averaged to obtain the feature stability score for all the feature selection algorithms. Table 10 depicts the stability scores obtained for the metaheuristics on three HAR datasets. It is evident from the table that ALO demonstrates profound stability and consistency over the other optimization algorithms in feature selection task for all three datasets.

## Conclusion

A 3D skeleton-based HAR problem has been addressed in this paper by incorporating compact spatio-temporal image encoding and evolutionary algorithm-based feature selection. The proposed method produces competitive results with the existing methods with lesser parameters by utilizing grayscale encoded images on three standard benchmark datasets. Results achieved on these datasets show the efficiency of the evolutionary optimization algorithm for feature selection. However, improvements can be made to the proposed framework in the future. In this work, we have used ALO for the feature selection step however, other more recent meta-heuristics optimization algorithms may also be used for this purpose. To further improve the robustness of the framework, we intend to extend the experimentation to more HAR datasets and possibly to other domains which use skeletal data in the future. We also plan to extend the experimentation involving different modalities of data (RGB video, depth, IR sequence data, etc.) for providing additional information to the classification framework, in order to achieve better as well as robust classification performance.

## Data Availability

No datasets are generated during the current study. The datasets analyzed during this work are made publicly available in this published article.
